# Thermoelectricity of near-resonant tunnel junctions and their relation to Carnot efficiency

**DOI:** 10.1038/s41598-021-81466-3

**Published:** 2021-01-21

**Authors:** Matthias A. Popp, André Erpenbeck, Heiko B. Weber

**Affiliations:** Department Physik, Friedrich-Alexander-Universität Erlangen-Nürnberg, Staudtstr. 7, 91058 Erlangen, Germany

**Keywords:** Materials science, Condensed-matter physics, Materials for devices, Materials for energy and catalysis, Nanoscale materials, Nanoscience and technology, Nanoscale devices, Nanoscale materials, Physics, Applied physics, Condensed-matter physics, Electronics, photonics and device physics, Quantum physics, Statistical physics, thermodynamics and nonlinear dynamics

## Abstract

We present a conceptual study motivated by electrical and thermoelectrical measurements on various near-resonant tunnel junctions. The squeezable nano junction technique allows the quasi-synchronous measurement of conductance *G*, *I*(*V*) characteristics and Seebeck coefficient *S*. Correlations between *G* and *S* are uncovered, in particular boundaries for *S*(*G*). We find the simplest and consistent description of the observed phenomena in the framework of the single level resonant tunneling model within which measuring *I*(*V*) and *S* suffice for determining all model parameters. We can further employ the model for assigning thermoelectric efficiencies $\eta$ without measuring the heat flow. Within the ensemble of thermoelectric data, junctions with assigned $\eta$ close to the Carnot limit can be identified. These insights allow providing design rules for optimized thermoelectric efficiency in nanoscale junctions.

## Introduction

Thermoelectric transport i.e. the unified consideration of heat and charge transport bears intrinsic correlations. The most famous among them is the Wiedemann–Franz law^1^, which connects heat conductivity and electrical conductivity in linear response, not only in the ohmic regime but also in nanoscale metallic junctions^2^. At functional interfaces with a sharp voltage and temperature drop (*V*,$\Delta T=T_H-T_C$) and nonlinear transmission function $\tau (E)$, however, it looses validity. There, optimum thermoelectric power conversion of the electronic system is achieved for Dirac-delta like $\tau (E)$^3^. This picture has been refined: in the presence of finite heat conductance by other channels (e.g. phonons, photons), boxcar functions promise maximum efficiency at finite power^4^.

Here, we focus on resonant tunneling^5,6^. It comprises essential electronic aspects for charge transport through quantum dots and, in particular, molecular junctions. A recent summary on thermoelectricity of the latter is given in^7,8^. There, the junction conductance *G* ranged from $10^{-5}\ldots 10^0\;G_0$ (with the conductance quantum $G_0=2h^2/e$) and corresponding Seebeck coefficients $S=- V_{th} / \Delta T$ (also termed thermopower, with thermovoltage $V_{th}$) are typically below 30 $\mu \mathrm {V}/\mathrm {K}$. This is, as it will turn out in this paper, a rather low value. When in addition electrostatic gates are present, parameter sets could be tuned to provide higher *S*^9,10^. Experiments on thermoelectricity of (resonant) quantum dot devices were carried out at temperatures of a few Kelvin and below^11–14^. We present experiments on resonant tunnel junctions/molecular junctions and theory that relate *G* and *S* and address the question whether correlations and/or design rules can be recognized.

**Figure 1:**
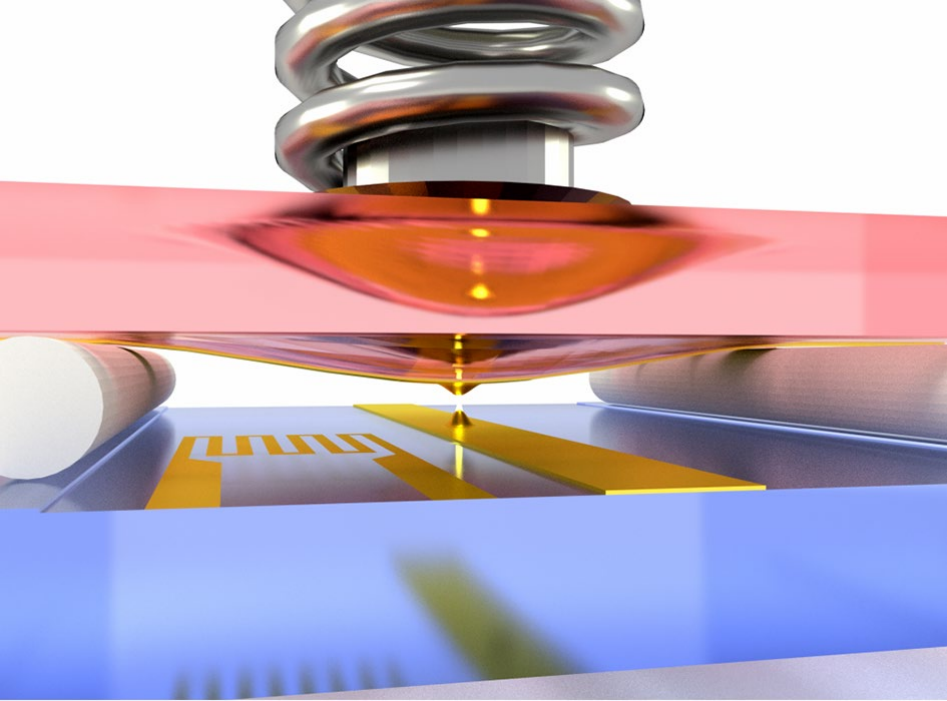
Scheme of the squeezable nanojunction (SNJ). Two SiC chips with electrodes are placed in a sandwich configuration. The ultra-stable distance between the electrodes is adjusted via a piezo-spring mechanism^15^. Chip temperatures $T_H$ and $T_C$ are monitored via on-chip resistance thermometry.

The recent development of squeezable nanojunctions (SNJ)^15^ gives access to such phenomena. We started our investigation with metallic junctions at various distances. In a next series of experiments, we studied junctions with added near-resonant states. The latter were introduced by molecules, nanoparticles and even unspecified contamination states that are sporadically observed in such experiments. These near-resonant configurations commonly enhanced the thermoelectric effects in a broad conductance range ($10^{-4}\ldots 10^0\;G_0$). The SNJ technique allows due to its extreme stability an immediate comparison of *S* and *G*, along with the additional information provided by the full *I*(*V*) of every single configuration, i.e. for the very same atomistic structure^15^. We identify so far obscured correlations between these quantities.

**Figure 2:**
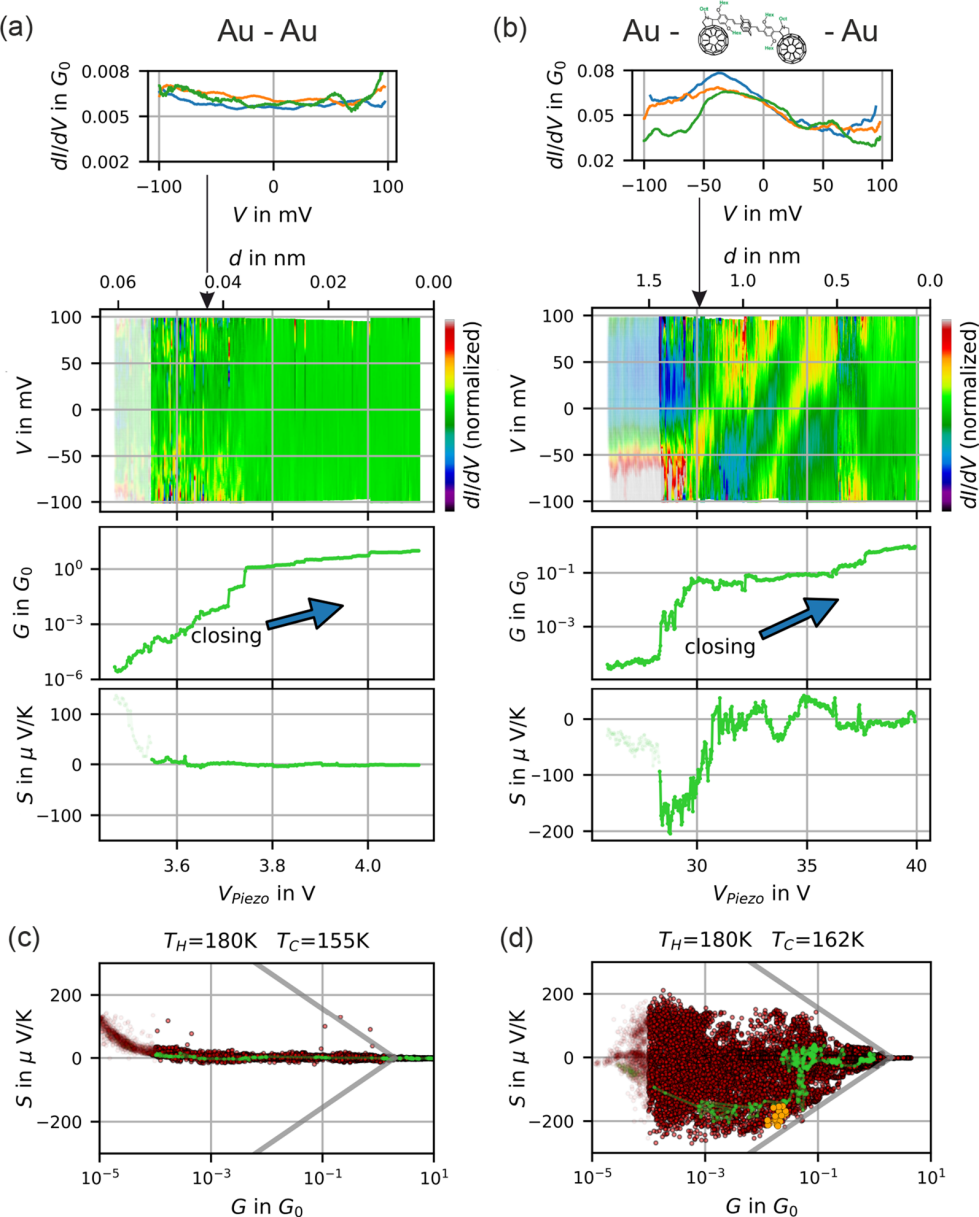
Electric and thermoelectric characterization. (**a**, **b**) Show example $\mathrm {d}I/\mathrm {d}V$ curves, shape evolution of $\mathrm {d}I/\mathrm {d}V$ (color coded, every $\mathrm {d}I/\mathrm {d}V$ is normalized to it’s mean value), conductances *G* and Seebeck coefficients *S* measured during mechanical variation of the junction’s distance (here: closing). (**a**) A bare gold-gold junction and (**b**) a junction with fullerene end-capped molecules^16,17^ (SI) applied to the electrodes. (**c**, **d**) Show correlations of *S* and *G* using the data of the whole ensembles enclosing 21 (27) opening and closing cycles with a total of 14431 (25519) $\mathrm {d}I/\mathrm {d}V$ and *S* measurements. Green dots correspond to the data in (**a**, **b**). Orange dots mark a sub-ensemble with high conversion efficiency. Gray lines are eye guides which separate $G-S$ pairs from exclusion areas.

## Experimental observations

We measure individual *I*(*V*) curves during stepwise opening and closing the nanojunction. During the experiment, the temperature difference $\Delta T$ is constantly applied. Figure 2(a) displays such a sequence during a closing cycle of a metallic junction (tunnel distance is reduced stepwise), with normalized $\mathrm {d}I/\mathrm {d}V$ represented in color code (divided by mean value). This representation puts emphasis on the evolution of the shape, but suppresses the exponential increase of conductance. From *I*(*V*) data *G* can be derived, yielding closing curves similar to previous nanojunction experiments. Immediately after each *I*(*V*) measurement, *S* is recorded such that each $G-S$ pair can be assigned to the same atomistic structure. This statement is supported by the continuous evolution of both quantities upon changing the distance. We define a data set as a pair of *I*(*V*) and *S*. About 20,000 of such data sets (20–30 opening/closing cycles) are considered as an experimental ensemble^18^. Figure 2(c) shows a full ensemble of $G-S$ data pairs, each of which is represented as a red dot.

The left and right column in Fig. 2 show data recorded in two independent experiments, representing the full range of phenomena that we observe. We first describe pure gold samples under high vacuum conditions (Fig. 2a,c). $\mathrm {d}I/\mathrm {d}V$ curves are approximately constant, the Seebeck coefficient for $G>G_0$ i.e. in the metallic regime is below 10 $\frac{\mu \mathrm {V}}{\mathrm {K}}$, the overall observations are in full agreement with previous measurements including step-wise reduction of conductance, pronounced plateaus at $G=G_0$^6,19^ and Seebeck coefficients as described by^20,21^. Below $G_0$ i.e. in the tunneling regime we find slightly enhanced thermopowers that scatter around $+\,1\;\frac{\mu \mathrm {V}}{\mathrm {K}}$ with a standard deviation of $6\,\frac{\mu \mathrm {V}}{\mathrm {K}}$. This represents an upper bound for the measurement error in the considered conductance range. The electronic noise should be even less since structural reconfiguration noise of the metallic junction is included in the data. Below $10^{-4}\;G_0$ deviations due to experimental artifacts were observed, more precisely a voltage that results from finite offset-currents of the voltage amplifier, which cannot fully be compensated for. From the measurement we can derive $\approx$ 1 pA for our setup.

The right column in Fig. 2 shows data obtained with intentionally added molecules. This specific species was used previously for single-molecule investigations at very low temperatures^16,17^ (also Fig. S1 (a), the molecule is depicted in Fig. S9). Here, the molecule was investigated in the intermediate range between stretched metal-molecule-metal junctions and metallic contact, i.e. the measurements cover a regime of strongly varying microscopic variations of the junction. Figure 1(b) shows data of a selected closing curve with resonances shifting through the Fermi level. Direct evidence is given by peaks in $\mathrm {d}I/\mathrm {d}V$ continously shifting their position from negative to positive bias voltages upon closing the junction (see Fig. 2(b), S5–S7 for near-resonant peaks and^15^). Additionally, closing curves of both *G* and *S* are shown. Note that highly structured $\mathrm {d}I/\mathrm {d}V$ coincide with high values for *S*. The corresponding $G-S$ data of the full ensemble are displayed in (d). For this (near-) resonant tunneling case, a qualitatively different picture is found: the Seebeck coefficient covers a wide span of values up to and even beyond 200 $\upmu \mathrm {V/K}$. This is more than an order of magnitude larger than in the (flat) tunneling regime (and our measurement error). It should be mentioned that no further selection or filtering was applied. The data explore large areas of the $G-S$ manifold like if there was no correlation. However, there are clear exclusion areas; their boundaries are indicated by gray solid lines as guide to the eyes, and are kept identical in all $G-S$ plots throughout this manuscript. We emphasize two unexpected findings uncovered by this plot: first, the Seebeck coefficients in the near-resonant tunneling regime are significantly higher than reported in earlier ungated single-molecule studies^7,8^. Second, *S* is limited by boundaries. It turns out that this physics, which will be defined more accurately by a theoretical ensemble analysis (vide infra), is not specific to the chosen molecule: Very similar findings could be obtained using nanoparticle^22^ junctions and unspecified contamination states (Fig. S1 (b),(d)). The latter are sporadically observed in molecular electronics, where on nominally clean nanocontacts, in particular upon voltage pulses, *I*(*V*) characteristics occur that may easily be confused with a molecular junction. It turns out that this similarity holds also for the thermoelectric characterization. The generality of the observation is why we emphasize in this paper on the general concept of near-resonant tunneling rather than on the properties of one specific molecule.

Figure 2(c) and (d) represent two limiting cases of our observed ensembles: whereas in Fig. 2(c) (pure gold) the data accumulate close to the $S=0$ axis, the data in (d) (with molecules providing resonant tunneling behavior) show a broader spread of *S* values. Further experiments delivered ensembles that were bouncing in between both cases (in particular for nominally metallic junctions, unspecified contaminations occured only sporadically). Further ensembles did not fill the full accessible part of the $G-S$ manifold, presumably because of incomplete sampling within the finite measurement time, or because of inherent asymmetries associated to the molecule/nanodot chosen. More data can be seen in SI. While we focus on the ensembles, a plethora of individual *I*(*V*) characteristics is not considered here in detail, but are available as raw data in^18^

**Figure 3:**
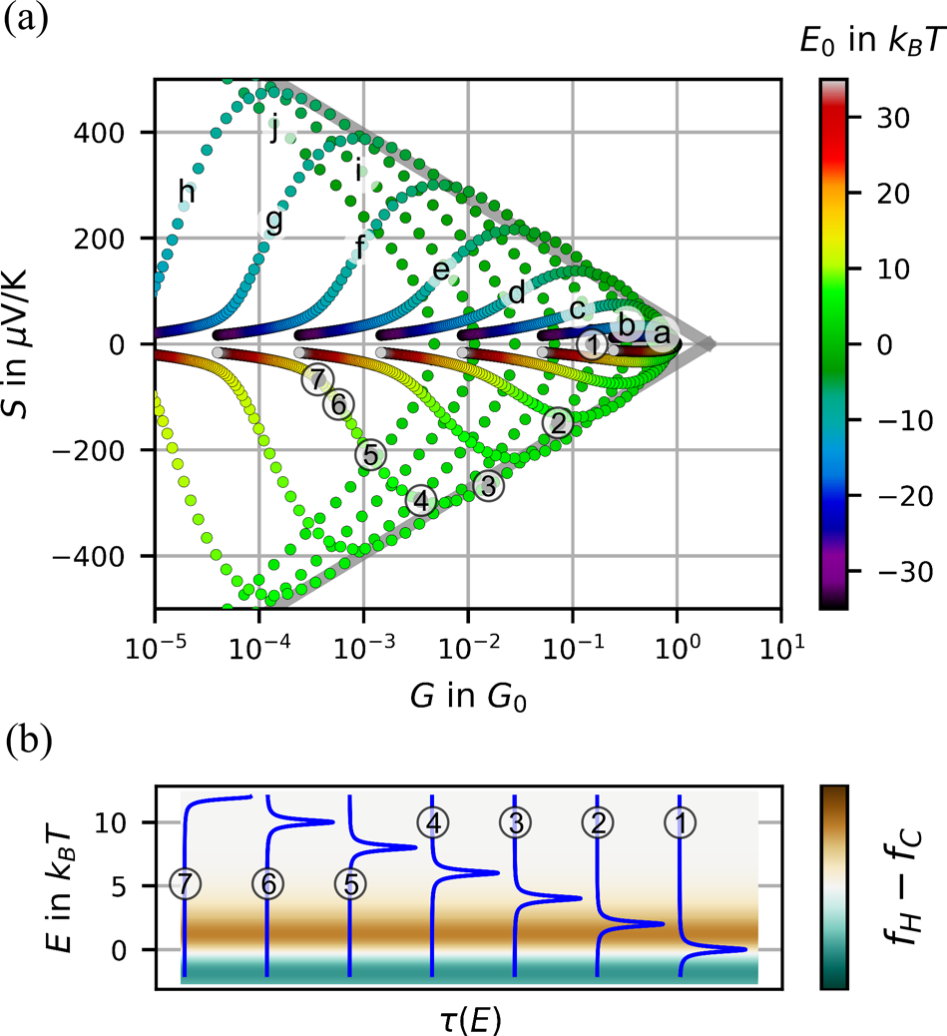
Conductance and Seebeck coefficient in the resonant level model. (**a**) Seebeck coefficients and conductances calculated within the resonant level model. The trajectories a-j are curves of constant $\Gamma$, varied logarithmically from $\Gamma =10\,k_BT$ to $\Gamma =3\cdot 10^{-3}\,k_BT$. Within each trajectory the position of the energy level $E_0$ was varied in equidistant steps indicated by the color scale. The gray lines are the very same eye guides as in Fig. 2 which mark the boundaries to the excluded area. (**b**) Transmission functions $\tau (E)$ along the trajectory with $\Gamma =1.1\cdot 10^{-1}\; k_BT$. The labels 1–7 correspond to the labels in (**a**).

## Theoretical analysis

In an effort of finding the simplest valid description of the experimental results, we discuss them in the framework of the resonant tunneling model within the Landauer–Büttiker transport picture. Obviously, this is a simplification of the problem, as it reduces a plethora of different atomistic structures to an ensemble of only few parameters. It further disregards the influence of vibrational and interaction effects. As we will see, despite its simplicity it provides access to understanding the interplay of thermoelectric quantities, in particular the findings displayed in Fig. 2^2,6,7^. We choose a Lorentz-shaped transmission function

1 $$\begin{aligned} \tau (E)=\frac{4\Gamma ^2}{(E-E_0)^2+4\Gamma ^2} \end{aligned}$$

at energy $E_0$ and width $\Gamma$. The latter reflects broadening due to coupling to the leads, which we assume to be symmetric for simplicity. In this picture *G* and *S* are calculated as

2 $$\begin{aligned} G= & {} \frac{2e^2}{h}\int -\frac{\partial f}{\partial E}\left( \frac{T_H+T_C}{2},E\right) \tau (E)dE, \end{aligned}$$

3 $$\begin{aligned} S= & {} \frac{1}{G} \frac{-2e}{h}\int [f(T_H,E)-f(T_C,E)] \tau (E)dE \end{aligned}$$

with the Fermi function $f(T,E)=1/(\mathrm {exp}(\frac{E-\mu }{k_BT}+1))$. We sample the parameter space by varying $E_0$ and $\Gamma$ for fixed $T_H$ and $T_C$. We choose a representation of *S* vs. $\log (G)$, motivated by the experiment. The result is shown in Fig. 3, where we choose ten different trajectories of constant $\Gamma$ (logarithmically equidistant) and vary $E_0$, the latter can be recognized by its color code. The theoretical ensemble that is sketched by the trajectories has a rounded arrow-head shape. We start the discussion with the case $E_0=E_F=0$ (resonant case, $\textcircled {1}$ in Fig. 3(b)), at which the fully symmetric Fermi distribution of electrons along with the symmetric transmission function of the level results in zero thermopower. Note that the conductance differs strongly from unity ($G\ne G_0$) when $\Gamma <k_BT$. When however lifting $E_0$ above the Fermi-energy, such that the Lorentz resonance is still within the thermal window (near-resonant case $\textcircled {2}$, $\textcircled {3}$), the Seebeck coefficient rises with $E_0$ and reaches remarkably high values, fully compatible with the experimental values. Beyond position $\textcircled {4}$, when the Lorentz resonance moves far out of the thermal window *S* decreases again (off-resonant case $\textcircled {5}$–$\textcircled {7}$). In this limit the quadratic roll-off of the Lorentzian gives the dominant contribution in Eq. (3). This overall behavior is fully symmetric for positive and negative $E_0$. Sampling the full $E_0,\; \Gamma$ parameter space, the exclusion areas observed in experiments are recovered without any further assumptions. The boundaries are formed by two enveloping lines which are identical with the gray lines in Fig. 2. A closer look reveals, however, that the envelope is slightly curved, the gray straight line is therefore rather a guide to the eye. Notably any $G-S$ data pair between the envelopes can be generated. This finding is robust when the assumption of symmetric coupling to the left and right electrode is lifted: then the overall pattern is maintained but shifted towards lower conductances (the exclusion area is not violated by this shift). Similarly when in a simple gedanken experiment two near-resonant tunneling paths in parallel form the junction, *S* is the same as for a single one, but the conductance *G* is doubled. This would then enter into the excluded area, which would, however, be barely visible on the logarithmic scale. We conclude that, despite its simplicity, the single resonant level model is suited to describe the experimental correlations between *S* and *G*. Although the fact that thermopower depends on level position and coupling is thus well known, the boundaries of the $G-S$ manifold of Fig. 3(a) had hitherto not been shown.

The shape of the chosen trajectories in Fig. 3 is not purely artificial. As an anecdotic example we highlighted one trajectory in Fig. 2(d) in green color, which corresponds to the closing curves in (b). In the range of $V_{Piezo} = 28. \;31$ V the energetic position of the resonance moves towards the Fermi energy accompanied by *G* increasing from $10^{-3}\ldots \,10^{-1}\;\; G_0$ and S varying between $-\,200\ldots \;0\;\; \mu \mathrm {V}/\mathrm {K}$. This may be explained by the continuous evolution of the all-important local environment of the molecule (electrostatic, strain, shape^10,23,24^).

**Figure 4:**
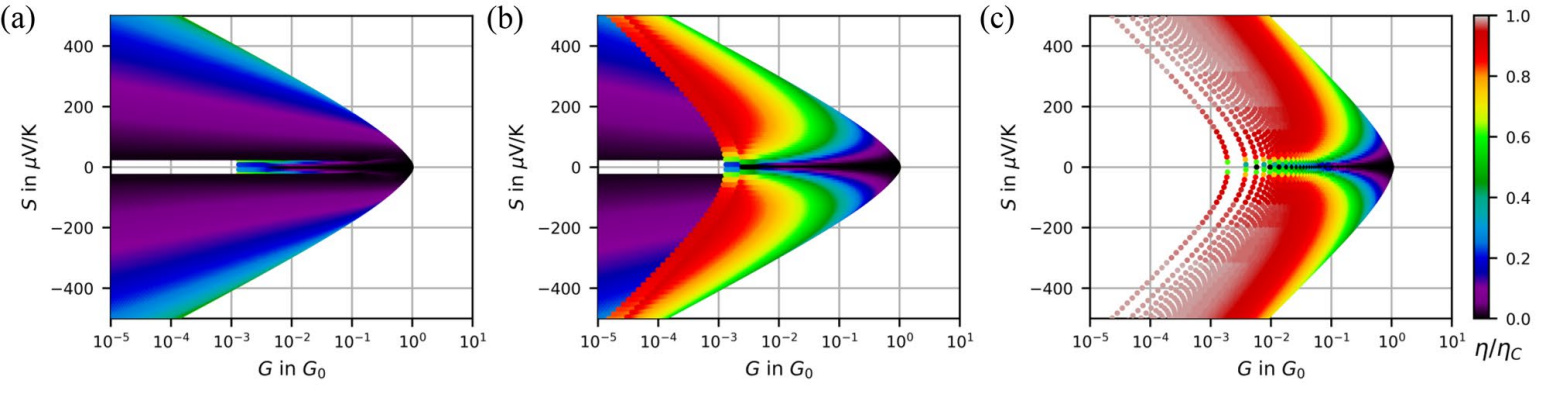
Heat conversion efficiency $\eta$ of the electronic system calculated with respect to Carnot-efficiency $\eta _C$. (**a**, **b**) are calculated within the resonant level model. In (**a**) the off-resonant regime is in the foreground whereas in (**b**) the near-resonant regime is visible. (**c**) rectangular transmission function for comparison. Note that here also the excluded area differs.

In any case high Seebeck coefficients coincide with near-resonant features in the $\mathrm {d}I/\mathrm {d}V$. Note that this detailed description along with an in-depth analysis of the resonant tunneling parameters (SI section 9) is accessible only because of the outstanding capabilities of the SNJ technique (stability and synchronous measurements of *G* and *S*). Otherwise fluctuations in $E_0$ would appear as fluctuations of *G* and *S*.

## Implications

By design, the SNJ technique is not suited for measuring the heat current, because the SiC chips are in thermal contact even when the electrical tunnel junction is open. However, having identified a suitable model of thermoelectrical transport through near-resonant tunneling states, we further extend our studies to its properties and implications. The Seebeck coefficient is often considered to be proportional to temperature *T* (Mott formula). In the model under investigation this is recovered only in the off-resonant case. When however the resonance is closer to the Fermi level (near-resonant case), where $\tau (E)$ is strongly curved, the Mott assumptions are not fulfilled. *S* can then be strongly nonlinear in *T* (it remains, however, a linear response to $\Delta T$). Further the Wiedemann-Franz law can be rediscovered in selected areas of the $G-S$ pattern: It is valid in the off-resonant case (position $\textcircled {7}$ and beyond), where both $\tau (E)$ and $\partial \tau (E)/\partial E$ are essentially constant at $E=E_F$. Next to the envelope $\textcircled {3}$, the Wiedemann-Franz ratio delivers the Lorentz number (see SI).

Notably, the $G-S$ envelope remains untouched by variations of *T*. This can be understood by regarding the scaling behavior of the three energy scales $k_BT$, $E_0$, $\Gamma$. Upon multiplication of all three quantities with a common constant factor, the resulting *G* and *S* values are untouched, as well as the $G-S$ pattern, underscoring the generality of the concept.

Next to large Seebeck coefficients and their boundaries, the heat conversion efficiency is of fundamental interest. It quantifies how much electrical power $P_{el}$ can be generated with respect to the invested heat flux $\dot{Q}_{in}$ necessary to maintain $T_H$:

4 $$\begin{aligned} \eta (V)= \frac{P_{el}(V)}{\dot{Q}_{in}(V)}=\frac{-V\cdot I(V)}{\dot{Q}_{in}(V)} \end{aligned}$$

in the bounds $0<V<V_{th}$ with

5 $$\begin{aligned} I(V)= & {} \frac{-2e}{h}\int \left[ f(T_H,E+\frac{eV}{2})-f(T_C,E-\frac{eV}{2})\right] \tau (E)dE, \end{aligned}$$

6 $$\begin{aligned} \dot{Q}_{in}(V)= & {} \frac{2}{h}\int (E+\frac{eV}{2})\left[ f(T_H,E+\frac{eV}{2})-f(T_C,E-\frac{eV}{2})\right] \tau (E)dE. \end{aligned}$$

Here, the voltage is assumed to drop symmetrically across the junction i.e. the electrochemical potential of the hot lead is assumed to be lowered by $\frac{eV}{2}$ and vice versa for the cold lead. As $\dot{Q}$ is not available in our experiment, we choose to calculate it from the very same transmission function. A similar procedure has been used by^13,14^ for quantum dots (energy scales 100 times smaller).

The maximum efficiency for given $\tau (E)$ is then calculated by numerically maximizing $\eta$ with respect to *V*. We normalize the efficiencies with respect to the Carnot efficiency $\eta _C=\frac{T_H-T_C}{T_H}$ that is the highest possible efficiency in accordance with the second law of thermodynamics. Figure 4(a) and (b) show color-coded numerical results, separately plotted for parameter regions corresponding to the off-resonant and the near-resonant case, respectively. It becomes immediately visible that efficiencies close to the Carnot limit (red color) are only possible in the near-resonant case (in accordance with^25–28^). There the transmission is dominated by a delta-peak like $\tau (E)$, meaning that the integrands in equations (5) and (6) are evaluated only at $E_0$^3,4^. The envelope has again special features: here, the efficiency is $0.5 \;\eta _C$ when being sufficiently distant from the arrow head tip. Such astonishingly high efficiencies were reached by our experiments. Notably these values appeared without targeted design of the molecule, and even in unspecified contamination states. For a better classification, we compare the $G-S$ plot for resonant junctions with boxcar transmission profiles that are the profiles that are expected to be best in efficiencies^4^, cf. Fig. 4(c). Indeed high efficiencies can be found at significantly higher conductance values which is favorable for thermoelectric heat conversion at high power output. Remarkable is, though, the very similar overall pattern, even if the boundaries are shifted outwards. This underscores that the sharper the transmission function is, the better the efficiencies can be expected.

However, when connecting the implications of the model with realistic experiments, further contributions should be considered that reduce this efficiency: (i) an electronic contribution with flat $\tau (E)$^15^ that provides an offset conduction $G_{off}$ in tunneling experiments, enclosing unspecified spectrally broad tunneling channels (electronic background transparency) and (ii) a vibrational heat conductance $G_{th,vib}$ in addition to the electronic heat conduction. Both do not contribute to thermovoltage. But these electronic terms are accessible by analyzing the full data set including *I*(*V*) out of which we can determine $E_0$, $\Gamma$, $\alpha$ and $G_{off}$ (see SI,^15,29^). Evaluating a subensemble (orange dots in Fig. 2(d)), we find $\eta _{res}=0.5\;\eta _C$ for the resonance-only case which leads to a thermoelectric figure of merit $ZT_{res}\approx 8$. When $G_{off}$ is included, it reduces to $\eta _{el}=0.3\;\eta _C$, ($ZT_{el}\approx 2.5$). Beyond the electronic contributions that are accessible by *I*(*V*) and *S* measurements, an additional loss channel can be provided by vibrational heat transport. For an estimate we choose the only experimentally determined value of vibrational heat conductance (measured with stretched alkane molecular junctions): $G_{th,vib}\approx 20\frac{\mathrm {pW}}{\mathrm {K}}$^30,31^ which finally leads to $\eta _{realistic}=0.1\;\eta _C$ ($ZT_{realistic}\approx 0.5$). These values are competitive compared to recent *ZT* record values^32–35^.

**Figure 5:**
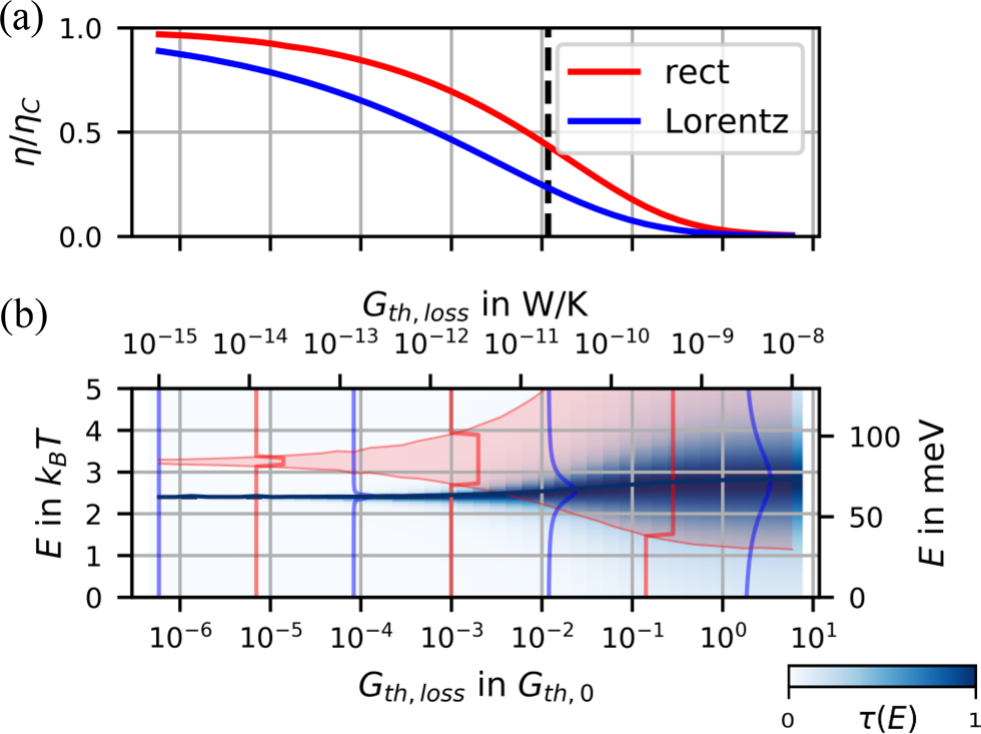
Efficiencies with optimized transmission functions. (**a**) Optimal values of the thermoelectric conversion efficiency for rectangular and Lorentz shaped transmission functions, plotted as a function of $G_{th,loss}$. (**b**) The underlying optimized transmission functions.This plot is temperature invariant when choosing units of $k_B T$ and the (electrical) thermal conductance quantum^36^ $G_{th,0}=\frac{2\pi ^2k_B^2T}{3h}$ (left and lower scale). The right and upper scale denominate values at room temperature (T = 300K).

On the first sight, an optimization of efficiency would reduce $$G_{th,vib}$$ and keep the electronic system untouched. Our model, however, gives access to further potential optimization: for a given $$G_{th,loss}$$ (including $$G_{th,vib} $$ and the thermal loss due to $$G_{off}$$), we search for parameters $$E_0,\;\Gamma $$ such that the efficiency is optimized. The numerical results are displayed in Fig. 5. In (a), the optimized $$\eta $$ is plotted both for the resonant tunneling model (blue) and a rectangular transmission window as a function of $$G_{th,loss}$$ in a broad range. Along with the results for the resonant level model we evaluated a hypothetical system with boxcar-type $$\tau (E)$$ which has shown to be the theoretically most efficient transmission function.

Hence, improvements targeting high efficiency interfaces should first ensure near-resonant electronic conditions, more explicitly $E_0\approx 2.5\;k_BT$ and $\Gamma \approx 0.5\;k_BT$. Favorable is, of course, the design of low heat conductance. In case $G_{th,loss}$ drops below $10^{-9}\,\frac{\mathrm {W}}{\mathrm {K}}$ (at 300 K), $\Gamma$ should be matched accordingly. Due to our numerical analysis this complicated design challenge becomes now better tractable: we lay down optimized parameters in a look-up table, see table S1.

It should again be clarified that heat flow and thermoelectric efficiencies can not be determined by our experiment. We nevertheless present these predictions because we found that the essence of thermoelectric correlations is adequately described by the near-resonant tunneling model, to the parameters of which our methods give access. For a critical test of heat conversion efficiencies, scanning tunneling microscopy should be invoked^30^.

## Conclusions

The recent development of the SNJ technique allows for quasi-synchronous measurement of *I*(*V*) characteristics and the Seebeck coefficient *S* of nanojunctions. The information of these experiments goes well beyond purely electrical measurements. Experimental ensemble analyses deliver boundaries of *G*(*S*) and can be described by the resonant level model. Within this model the boundaries are temperature independent, as opposed to the frequently used Mott approximation. An in-depth analysis of individual *I*(*V*) and *S* pairs gives access to model parameters of electronic snapshots during the evolution of nanojunctions in opening/closing cycles. The above sketched concept can be employed for predicting the electronic part of heat flow. Also the electronic part of the thermoelectric conversion efficiencies can be assigned. Without further ado, we find configurations that reach efficiencies remarkably close to the Carnot limit. The latter results demand for a critical experimental test with STM techniques.

## Methods

### Squeezable nano junction

The SNJ is composed of two silicon carbide (SiC) chips with gold electrodes and thermometers on top, placed face to face in a sandwich configuration see Fig. 1. Without external force, the chips are touching each other but the electrodes are not in electrical contact. By a piezo/spring mechanism this stack is compressed such that the minimum distance between the electrodes is controlled with extremely high stability and resolution^15^. The closing process is carefully controlled until reaching a metallic junction indicated by conductance of $G_0=1/12.9\,\mathrm{k}\Omega$ or higher. Subsequently the junction is reopened. The SNJ was mounted inside a vacuum vessel (high vacuum) on a cryostat.

### Electrical measurements

A coaxial relay switch was used to switch between *I*(*V*) and thermovoltage mode. In *I*(*V*) mode voltage is sourced by a data acquisition (DAQ) card (National Instruments, USB 6221) (linear ramps: 0-max–min-0). Current was preamplified by an I–V converter (Stanford Research Systems, SR570) and measured via DAQ card. Acquisition times of *I*(*V*) curves were 0.5 s. These short measurement times were chosen to make sure that successive *I*(*V*) curves can describe the same structures (*I*(*V*) measurement time < lifetime of junctions). In thermovoltage mode the relay was opened, creating open-contact conditions. Thermovoltages were amplified via a pre-amplifier (FEMTO, DLPVA) and digitized via DAQ card. Offset currents caused by the pre-amplifier were compensated using a source measure unit (Agilent, E5287A) in current source mode (offset current constant throughout measurement). This increased the trusted conductance range from $G>10^{-3} G_0$ to $G>10^{-4} G_0$ (offset current decreased from $\sim$ 10 pA to $\sim$ 1 pA). A circuit diagram and more detailed description can be found in SI.

### Temperature measurement

Temperatures of the sample holders were regulated (measured with Si-diode thermometers) in the measurements underlying Fig. 2 to 200 K and 150 K, respectively. The excellent heat conductivity of SiC guarantees that both chips have a defined temperature. Temperatures of both chips were constant throughout measurements and were measured via lithographically defined gold resistance thermometers on chip. These were calibrated during cooling down the cryostat. Low temperature measurement provides the advantage of increased contact stability compared to room temperature.

### Junction preparation

Figure 2(a, c): Electrodes are layers of 50 nm gold on top of a 5 nm titanium adhesion layer without further treatment. Figure 2(b, d): A $10^{-4}$ molar solution of fullerene end-capped molecules^16,17^ (SI) in CS$_2$ was drop-casted on one of the gold electrodes prior to measurement and immediately blow-dried with nitrogen. Junctions were pre-characterized by taking several *I*(*V*) characteristics in a voltage range of ${-}$ 0.7...0.7 V until strongly nonlinear curves were observed. This step was common to all measurements exhibiting high Seebeck coefficients. Ensemble measurements were carried out in a smaller voltage range of ${-}$ 0.1...0.1 V where the junctions are stable.

### Measurement protocol

Mount samples. Open/close junction in equidistant $V_{Piezo}$ steps. Measure *I*(*V*), $V_{th}$ and on-chip thermometer resistances consecutively between piezo steps. Repeat during following process. Cool down cryostat and use on-chip thermometer resistances and Si-diode temperatures for calibration. Heat up one chip introducing temperature difference. Pre-characterize junction and adjust piezo step width. Wait until stable conditions are reached ($\sim$ 0.5 h). Conduct ensemble measurement without changing parameters over night.

### Calculation methods

*dI*/*dV* curves were derived numerically from *I*(*V*): $\mathrm {d}I/\mathrm {d}V (V_{test})$ is the slope of a linear fit to the raw *I*(*V*) values with voltage in the interval $V_{test}\pm 20$ mV. Conductances *G* in Fig. 2 are obtained during the measurement^18^ by a linear fit in the interval ${-}$ 100 mV...100 mV; a re-analysis within the interval ${-}$ 10 mV...10 mV yields slightly smaller values of *G*. The difference is barely visible in the logarithmic representation of Fig. 2 and does not affect the conclusions.

## Supplemental Information

See Supplemental Information for extended data, calculations on the Wiedemann-Franz law, description of fit routine, a look-up table as a different representation of Fig. 5.

The full raw data underlying all figures in the main manuscript and SI are availible under^18^.

## Supplementary information

Supplementary Information
