# Supplementary material for: Thermoelectricity of near-resonant tunnel junctions and their relation to Carnot efficiency
Source: Sci Rep. 2021 Jan 21;11:2031. doi: 10.1038/s41598-021-81466-3 (PMC7820355; doi:10.1038/s41598-021-81466-3)
Supplement: Supplementary file 1 — Supplementary Information [file 41598_2021_81466_MOESM1_ESM.pdf]

Thermoelectricity of near-resonant tunnel junctions and their relation to Carnot efficiency – Supplemental Information –

Matthias A. Popp, André Erpenbeck, Heiko B. Weber

September 8, 2020

Contents

1 Extended data **3**

2 Formula collection and Wiedemann-Franz calculations **5**
2.1 Calculation of G and S 5
2.2 Calculation of η 5
2.3 Wiedemann-Franz law and the $G-S$ plot 6

3 Resonant level parameter determination from $I(V)$ data **7**

4 Optimized parameters for maximum efficiency at given loss heat conductivity **11**

5 Multiple parallel resonant tunneling channels **12**

6 Opening / closing curves and length estimation **13**

7 Molecules **18**

8 Electrical measurement circuit **18**

9 In-depth discussion of the anecdotic trajectory in FIG. 1 (b),(d) **20**

1 Extended data

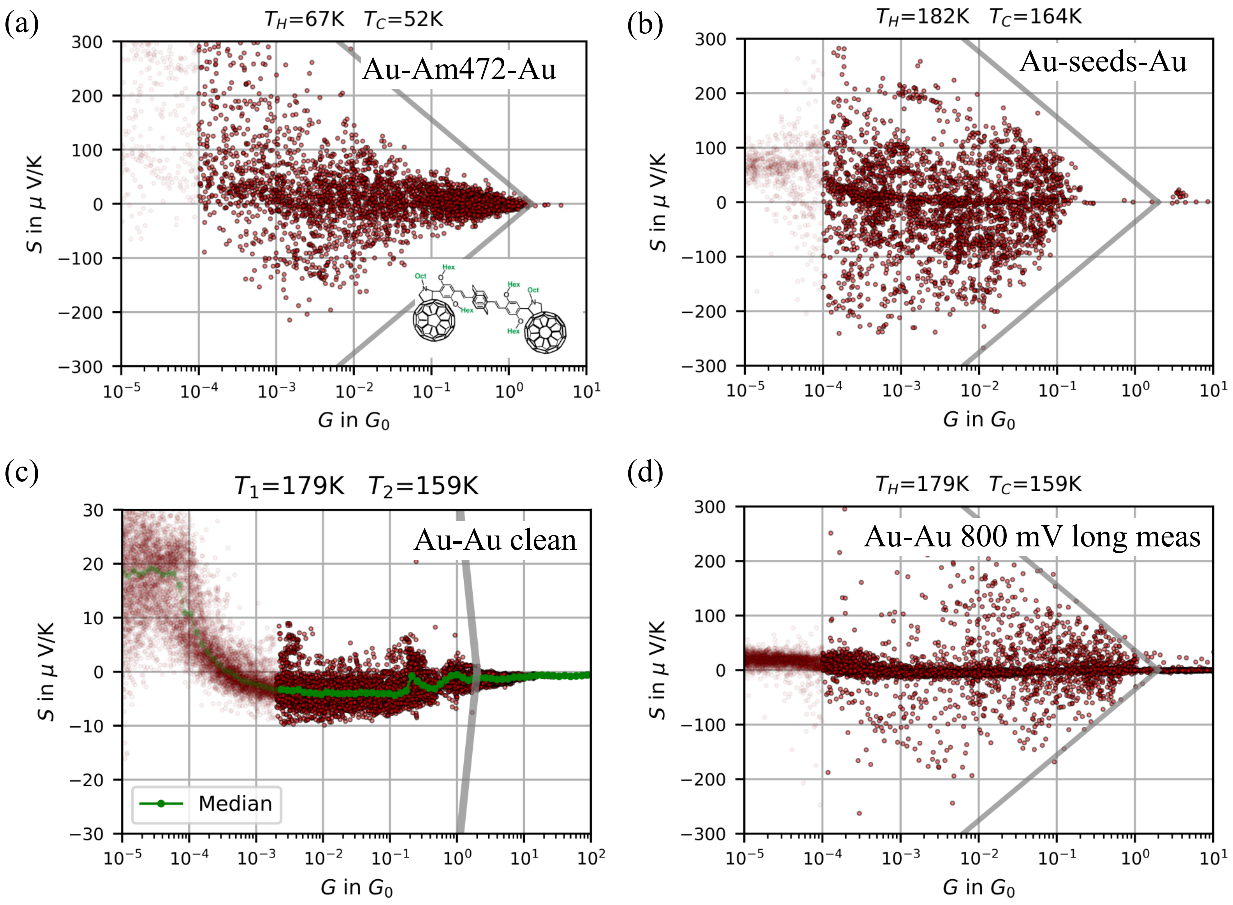

Figure S1: Extended data with varied gold-interface-gold systems.

Figure S1 shows more data sets measured following slightly varying routines compared to FIG. 1. of the main manuscript:

- **Figure S1 (a)**: The samples used for this measurement are the same samples as used for FIG. 1 (b), (d) of the main manuscript. A 10^{-4} molar solution of the molecule shown in the inset was dropcasted on one of the gold electrodes prior to measurement. Immediately after a droplet of solution was applied the sample was blowdried with nitrogen. During the whole measurement I(V) characteristics with $V_{peak} = 800$ mV as well as $V_{peak} = 100$ mV were recorded.

- **Figure S1 (b)**: A solution with CTAB stabilized gold nanoparticle seeds [1] was dropcasted on one of the electrodes prior to measurement. Immediately after a droplet of solution was applied the sample was blowdried with nitrogen. During the whole measurement I(V) characteristics with $V_{peak} = 800$ mV as well as $V_{peak} = 100$ mV were recorded.

- **Figure S1 (c)**: The sample pair with clean gold electrodes (50 nm gold with 5 nm Ti adhesion layer) was plasma-cleaned prior to measurement in O_2 Plasma. During the whole measurement I(V) characteristics only with $V_{peak} = 100$ mV were recorded. Seebeck coefficients below 10 μV/K were observed in the whole tunneling regime corresponding to metallic behavior.

- **Figure S1 (d)**: Measurement taken immediately after (c) with the same sample pair still mounted and cooled down. During the whole measurement I(V) characteristics with $V_{peak} = 800$ mV as well as $V_{peak} = 100$ mV were recorded. Seebeck coefficients in a -200..300 μV/K range were observed during a coherent period of 2h of the 18 h lasting measurement. The opening/closing distance (piezo voltage) was also increased during this period.

The same qualititive behavior including large S and boundaries in the $G-S$ plane is observed for different gold-interface-gold systems. We interpret this as a strong indication for the generality of the observations.

2 Formula collection and Wiedemann-Franz calculations

In this section formulae used for simulations are summarized. All calculations were carried out numerically using python scripts.

2.1 Calculation of G and S

Conductivity:

$$G = \frac{2e^2}{h} \int -\frac{\partial f}{\partial E} \left(\frac{T_H + T_C}{2}, E \right) \tau(E) dE \tag{1}$$

Thermocurrent (leads on same electrical potential, shorted electrodes)

$$I_{th} = \frac{-2e}{h} \int [f(T_H, E) - f(T_C, E)] \tau(E) dE \tag{2}$$

Seebeck coefficient (also termed thermopower)

$$S = \frac{I_{th}}{\Delta T G} \tag{3}$$

The resonant level model implies as transmission function [2]:

$$\tau(E) = \frac{4\Gamma_H \Gamma_C}{(E - E_0)^2 + (\Gamma_H + \Gamma_C)^2} \tag{4}$$

Which is simplified by assuming symmetric coupling $\Gamma = \Gamma_H = \Gamma_C$:

$$\tau(E) = \frac{4\Gamma^2}{(E - E_0)^2 + 4\Gamma^2}, \tag{5}$$

a Lorentzian function centered at E_0 which has a full width at half maximum of 4Γ.

2.2 Calculation of η

The voltage-dependent efficiency is

$$\eta(V) = \frac{P_{el}(V)}{\dot{Q}_{in}(V)} = \frac{-V \cdot I(V)}{\dot{Q}_{in}(V)} \tag{6}$$

in the bounds $0 < V < V_{th}$ with the voltage-dependent current

$$I(V) = \frac{-2e}{h} \int \left[f\left(T_H, E + \frac{eV}{2} \right) - f\left(T_C, E - \frac{eV}{2} \right) \right] \tau(E) dE \tag{7}$$

and the (voltage dependent) heat flow into the hot lead necessary to maintain T_H:

$$\dot{Q}_{in}(V) = \frac{2}{h}\int \left(E+\frac{eV}{2}\right)\left[f\left(T_H, E+\frac{eV}{2}\right) - f\left(T_C, E-\frac{eV}{2}\right)\right]\tau(E)dE \quad (8)$$

The efficiency of an E_0, Γ pair was obtained by numerically maximizing $\eta(V)$ with respect to V.

When including lossy channels a term $G_{th,loss}(T_H - T_C)$ was added to eq. (8)

2.3 Wiedemann-Franz law and the $G-S$ plot

The heat conductance (of the electronic system, at zero electrical current) was calculated using

$$G_{th} = \frac{2}{hT}\left(K_2 - \frac{K_1^2}{K_0}\right) \quad (9)$$

with

$$K_n = \int_{-\infty}^{\infty} \frac{\partial f}{\partial E}\tau(E)E^n \mathrm{d}E \quad (10)$$

[3].

Figure S2 shows simulations of the same parameter set as shown in FIG.3 of the main manuscript. Here, the color scale is given as $\log_{10}(L/L_0)$ Where

$$L = \frac{G_{th}}{GT} \quad (11)$$

with the Lorentz number $L_0 = \frac{\pi^2}{3}\left(\frac{k_B}{e}\right)^2$. This fraction relates the heat conduction of a given system to the heat conduction calculated within the Wiedemann-Franz law.

For the off-resonant case (a) we find that the heat conduction is generally higher than stated by the Wiedemann-Franz law. The more remote E_0 is from E_F the better the Wiedemann-Franz law is obeyed (red region close to the x-axis in Figure S4 (a)). Here, the behavior is dominated by the high-energy tail of $\tau(E)$ resulting in roughly constant $\tau(E)$ and $\mathrm{d}\tau(E)/\mathrm{d}E$ and therefore in the Wiedemann-Franz law (Sommerfeld expansion valid).

In the near-resonant regime depicted in Figure S2 (b) however less heat conduction than in the Wiedemann-Franz case is obtained. This corresponds to the finding that here the heat conversion efficiency is especially high. Also, at $G \approx G_0$ the Wiedemann-Franz law is obeyed. This can again be explained with flat $\tau(E)$, this time due to large broadening Γ.

Surprisingly, at the transition between the two regimes, i.e. at the boundaries of the allowed $G-S$ region where $S(G)$ is maximized, $L/L_0 = 1$ is valid. However for the generating parameters E_0, Γ kept constant, a variation in T is not described by $G_{th} \propto GT$.

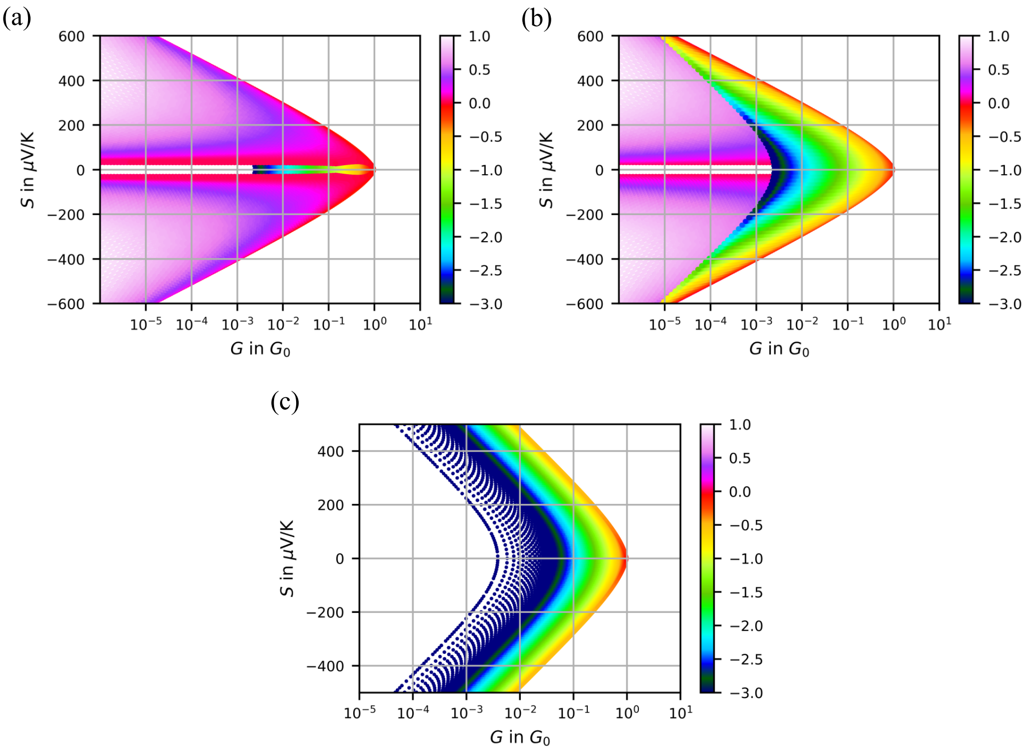

Figure S2: Wiedemann-Franz law and resonant level model. Color scales are expressed in units of $\log_{10}(L/L_0)$. In (a) the off-resonant regime of single resonant level transport is in the foreground. In (b) the near-resonant and resonant regime are visible. In (c) the results of an analogous calculation with rectangular $\tau(E)$ are shown

3 Resonant level parameter determination from $I(V)$ data

The applied fitting routine is mostly analogous to our previous publication [4]. We use the Landauer formalism in order to calculate the voltage dependent current $I(V)$:

$$I(V) = \frac{-2e}{h}\int[f(E+\alpha eV, T_H) - f(E-(1-\alpha)eV, T_C)]\tau(E)\mathrm{d}E \qquad (12)$$

α is a voltage division factor which describes the symmetry/asymmetry of the coupling of the scattering center to the leads. For $\alpha = 0.5$ the chemical potentials of both electrodes are shifted symmetrically when applying a voltage. $\alpha = 1$

would correspond to only shifting the chemical potential of the hot electrode, i.e. strong coupling of the scattering center to the cold electrode. As transmission function we use the Lorentz shape of the resonant-level model in combination with an additive constant

$$\frac{4\Gamma_H\Gamma_C}{(E-E_0)^2+(\Gamma_H+\Gamma_C)^2}+\frac{G_{off}}{G_0} \tag{13}$$

The parameter G_{off} models a parallel channel with constant transmission function which can be physically motivated by a non-resonant (background) tunneling channel. In previous analysis this has shown to improve the fit quality [4]. With respect to the heat conversion efficiency regarded in this paper G_{off} describes one of the main loss mechanisms which are important to find a realistic picture. By stating

$$\Gamma_H = (1-\alpha)\Gamma, \Gamma_C = \alpha\Gamma \tag{14}$$

we make a connection between the distribution of potentials and the symmetry of couplings (also see [2]). We fit this model with parameters E_0, α, Γ and G_{off} to the $I(V)$ and $\mathrm{d}I/\mathrm{d}V$ curves corresponding to the orange dots of FIG. 2. (b) of the main manuscript.

All integrals were evaluated numerically and fitting is performed using the *minimize()* routine of the python package LMFIT. Figure S3 shows examples of fitted curves. Fit parameters and derived efficiencies are summarized in table S1. Efficiencies were calculated according to the routine described in the main manuscript.

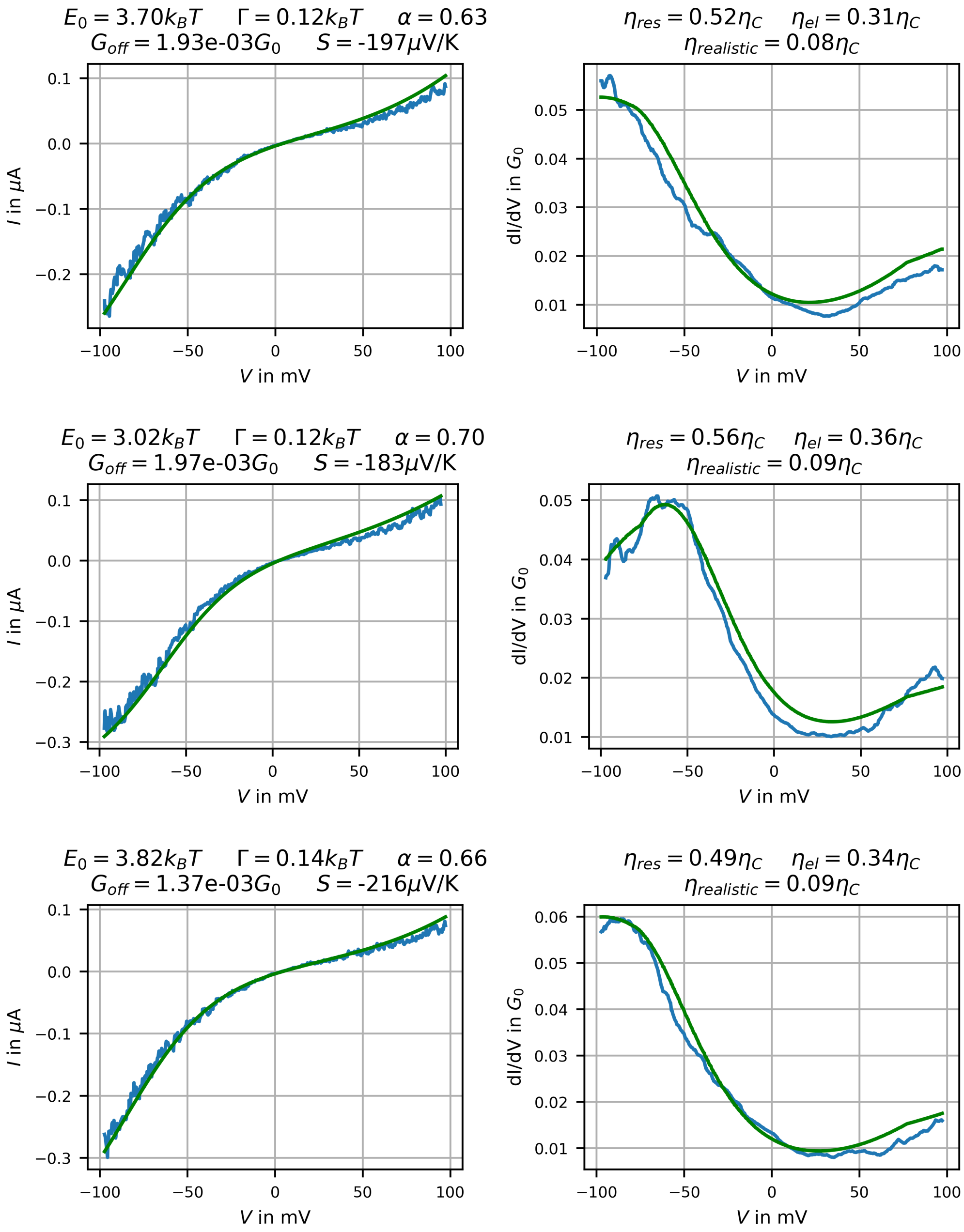

Figure S3: Measured $I(V)$ and $dI/dV(V)$ curves with fit of resonant level model. G-S pairs are marked orange in FIG. 2 (b) of the main manuscript.

Table S1: Resonant level parameters and efficiencies for the data subset marked with orange dots in FIG. 2 (b) of the main manuscript.

$G(G_0)$	$S(\mu V)$	$E_0(k_BT)$	$\Gamma(k_BT)$	α	$G_{off}(G_0)$	η_{res}/η_C	η_{el}/η_C	$\eta_{realistic}/\eta_C$
0.0141	-182	3.47	0.16	0.69	0.0017	0.49	0.35	0.10
0.0124	-176	3.96	0.22	0.66	0.0033	0.41	0.25	0.09
0.0130	-169	3.54	0.22	0.83	0.0001	0.43	0.42	0.10
0.0101	-153	3.42	0.17	0.77	0.0025	0.49	0.29	0.08
0.0116	-170	3.21	0.12	0.69	0.0001	0.55	0.53	0.11
0.0114	-154	3.72	0.14	0.70	0.0042	0.50	0.21	0.06
0.0086	-158	3.44	0.10	0.70	0.0028	0.57	0.26	0.06
0.0136	-184	3.27	0.13	0.70	0.0006	0.54	0.46	0.10
0.0100	-197	3.80	0.12	0.63	0.0023	0.52	0.27	0.07
0.0117	-205	3.81	0.14	0.61	0.0018	0.49	0.31	0.09
0.0115	-197	3.70	0.12	0.63	0.0019	0.52	0.31	0.08
0.0136	-201	3.55	0.13	0.65	0.0015	0.52	0.35	0.09
0.0137	-183	3.02	0.12	0.70	0.0020	0.56	0.36	0.09
0.0122	-184	4.04	0.14	0.66	0.0026	0.47	0.24	0.07
0.0065	-213	3.84	0.10	0.64	0.0015	0.55	0.30	0.06
0.0111	-213	3.84	0.14	0.71	0.0012	0.49	0.34	0.08
0.0133	-216	3.82	0.14	0.66	0.0014	0.49	0.34	0.09

4 Optimized parameters for maximum efficiency at given loss heat conductivity

Table S2: Optimized parameters for given loss thermal conductance $G_{th,loss}$. $\Gamma, E_0, \eta_{opt,Lorentz}$ are calculated (at 300 K) according to the resonant tunneling model with symmetric coupling. E_1,E_2 are boundaries of a rectangular transmission function resulting in $\eta_{opt,rect}$

$G_{th,loss}$ (pW/K)	Γ (k_BT)	$E_0(k_BT)$	$\eta_{opt,Lorentz}/\eta_C$	$E_1(k_BT)$	$E_2(k_BT)$	$\eta_{opt,rect}/\eta_C$
1.00E-03	1.20E-03	2.40	0.89	3.20	3.30	0.97
2.29E-03	1.82E-03	2.42	0.86	3.18	3.31	0.96
5.22E-03	2.74E-03	2.40	0.84	3.16	3.33	0.95
1.19E-02	4.15E-03	2.42	0.80	3.13	3.36	0.93
2.73E-02	6.26E-03	2.41	0.76	3.10	3.40	0.91
6.24E-02	9.45E-03	2.41	0.72	3.06	3.45	0.89
1.43E-01	1.43E-02	2.41	0.66	2.97	3.48	0.85
3.26E-01	2.15E-02	2.43	0.60	2.93	3.61	0.81
7.44E-01	3.23E-02	2.43	0.54	2.84	3.75	0.76
1.70E+00	4.84E-02	2.45	0.46	2.72	3.90	0.70
3.89E+00	7.21E-02	2.47	0.39	2.58	4.16	0.62
8.89E+00	1.06E-01	2.50	0.31	2.40	4.52	0.53
2.03E+01	1.55E-01	2.55	0.23	2.19	5.04	0.43
4.64E+01	2.19E-01	2.60	0.16	1.94	5.87	0.33
1.06E+02	2.96E-01	2.66	0.10	1.69	7.39	0.23
2.42E+02	3.77E-01	2.72	0.06	1.48	10.38	0.14
5.54E+02	4.45E-01	2.77	0.03	1.32	20.18	0.08
1.27E+03	4.92E-01	2.80	0.01	1.23	16.65	0.04
2.89E+03	5.18E-01	2.82	0.01	1.17	16.30	0.02
6.61E+03	5.31E-01	2.82	0.003	1.17	23.37	0.01

5 Multiple parallel resonant tunneling channels

Figure S4 shows simulation results with multiple parallel channels. The total transmission functions were assumed to be

$$\tau_{tot}(E) = \sum_{i}^{n} \tau_{E_{0,i},\Gamma_i}(E). \tag{15}$$

with $\tau_{E_{0,i},\Gamma_i}$ being a Lorentzian curve centered at $E_{0,i}$ with width Γ_i which were randomly chosen. If only few states are in parallel the findings obtained in the single resonant level model are mainly unchanged. However the boundaries in the S-G plane are violated in rare cases. For $n = 10..100$ the behavior changes more drastically. High S values are less probable and especially at high G deviations from the single resonant level model occur (see Figure S4).

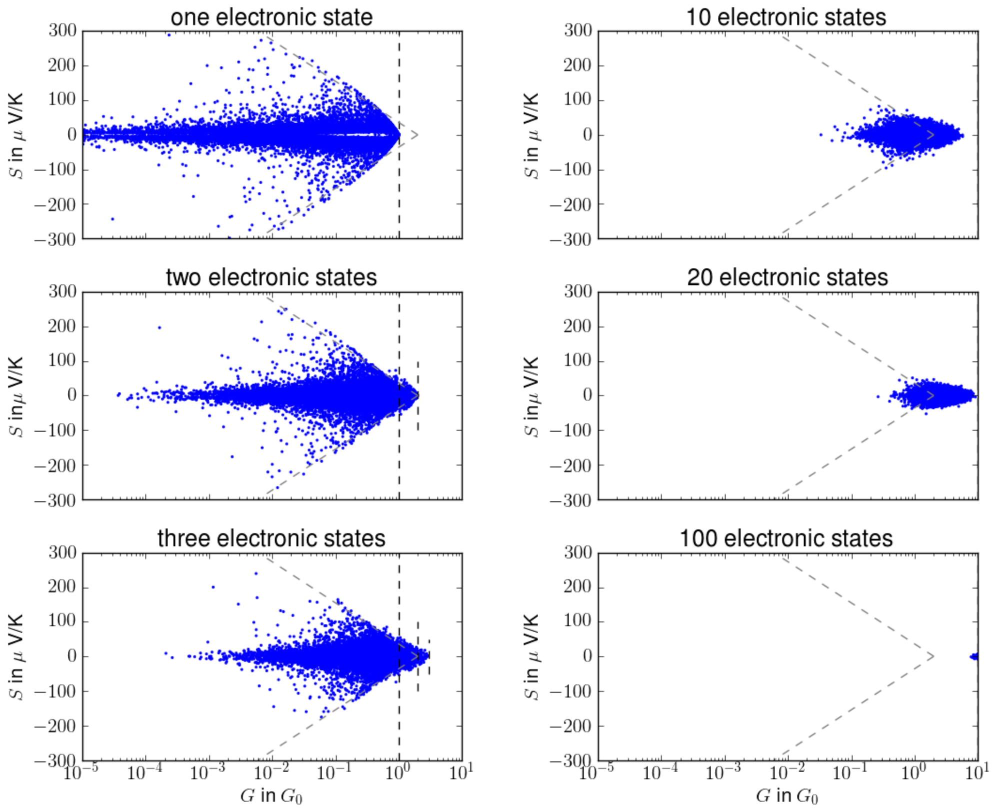

Figure S4: Simulated G-S values for n parallel channels with randomly chosen E_0, Γ

6 Opening / closing curves and length estimation

In this section we provide more exemplary opening/closing curves being subsets of FIG. 1 (d) of the main text. The whole ensemble comprises 27 opening/closing cycles out of which we show 4 exemplary ones in Figures S5 to S8. Opening/closing curves corresponding to all shown ensembles (main manuscript and SI) can be found in [5] in the form of raw data as well as image files.

Following [6] and using the exponential relation between tunneling current and distance

$$\frac{I(V,d)}{V} = K \cdot e^{-2d\sqrt{2m\phi/\hbar^2}} = K \cdot 10^{d/d_{dec}} \tag{16}$$

with K being a constant not used in the following, m the mass of an electron and the tunneling barrier $\phi = 5.4$ eV resulting in $d_{dec} = 1.37\,Å$, the distance variation needed to vary the conductivity by a decade in case of clean gold contacts. We use this to roughly estimate distance values d from V_{Piezo}.

Figure S8 shows a subset of FIG. 1(d) in which rupture of a presumably pure gold contact ($G \approx 10\ G_0$) is followed by clean exponential decay in the tunneling regime, accompanied with small thermovoltages and therefore tunneling between clean gold electrodes. We use this subset to perform the calibration described above and find a calibration factor of $\Delta d/\Delta V_{Piezo} = 1.29\,Å$/V.

An analogous procedure was performed for the data from FIG. 1(a), yielding a calibration factor of $\Delta d/\Delta V_{Piezo} = 0.1\,Å$/V. The difference is due to different geometries of the samples.

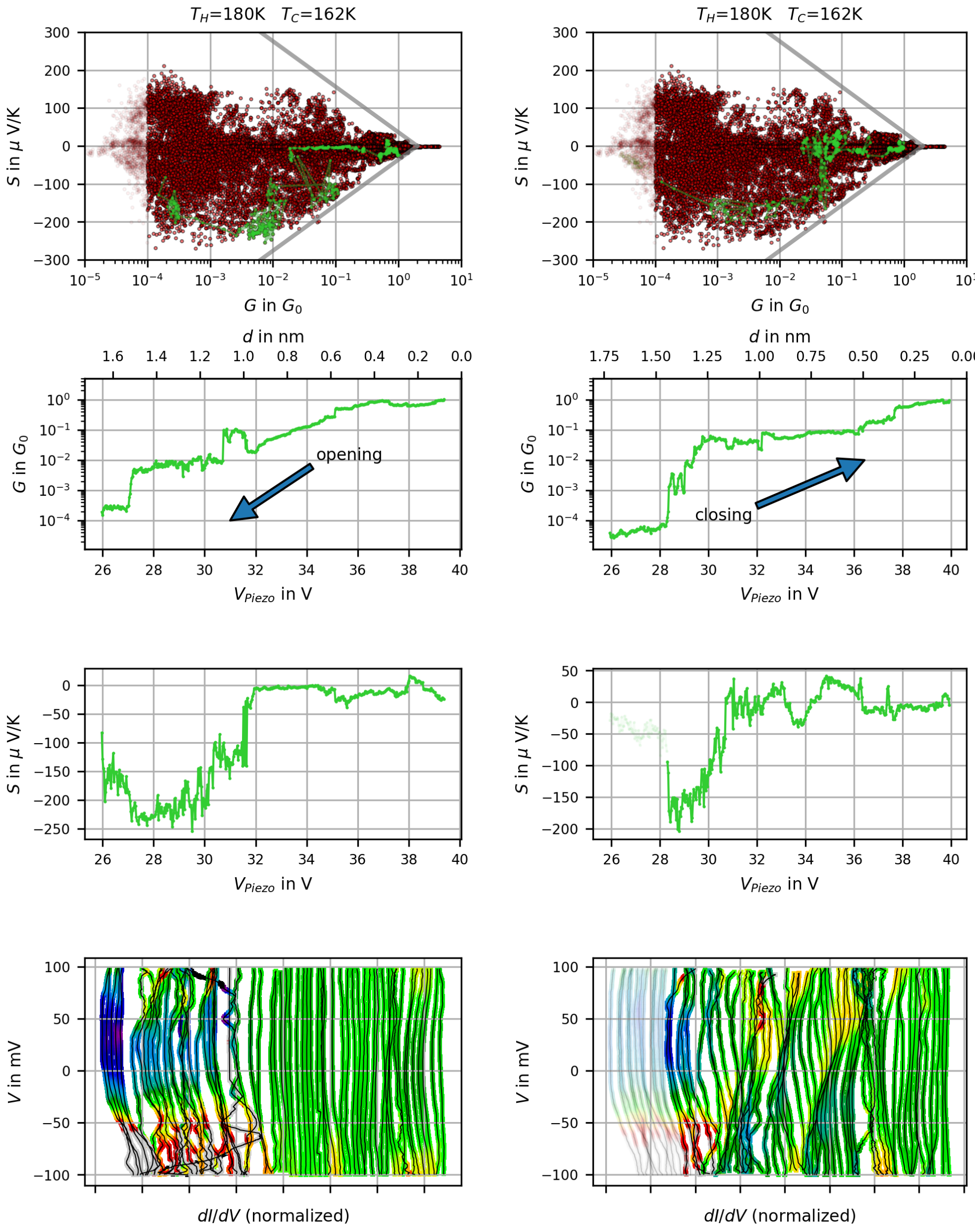

Figure S5: Exemplary opening/closing curves. $n = 3800..4715$

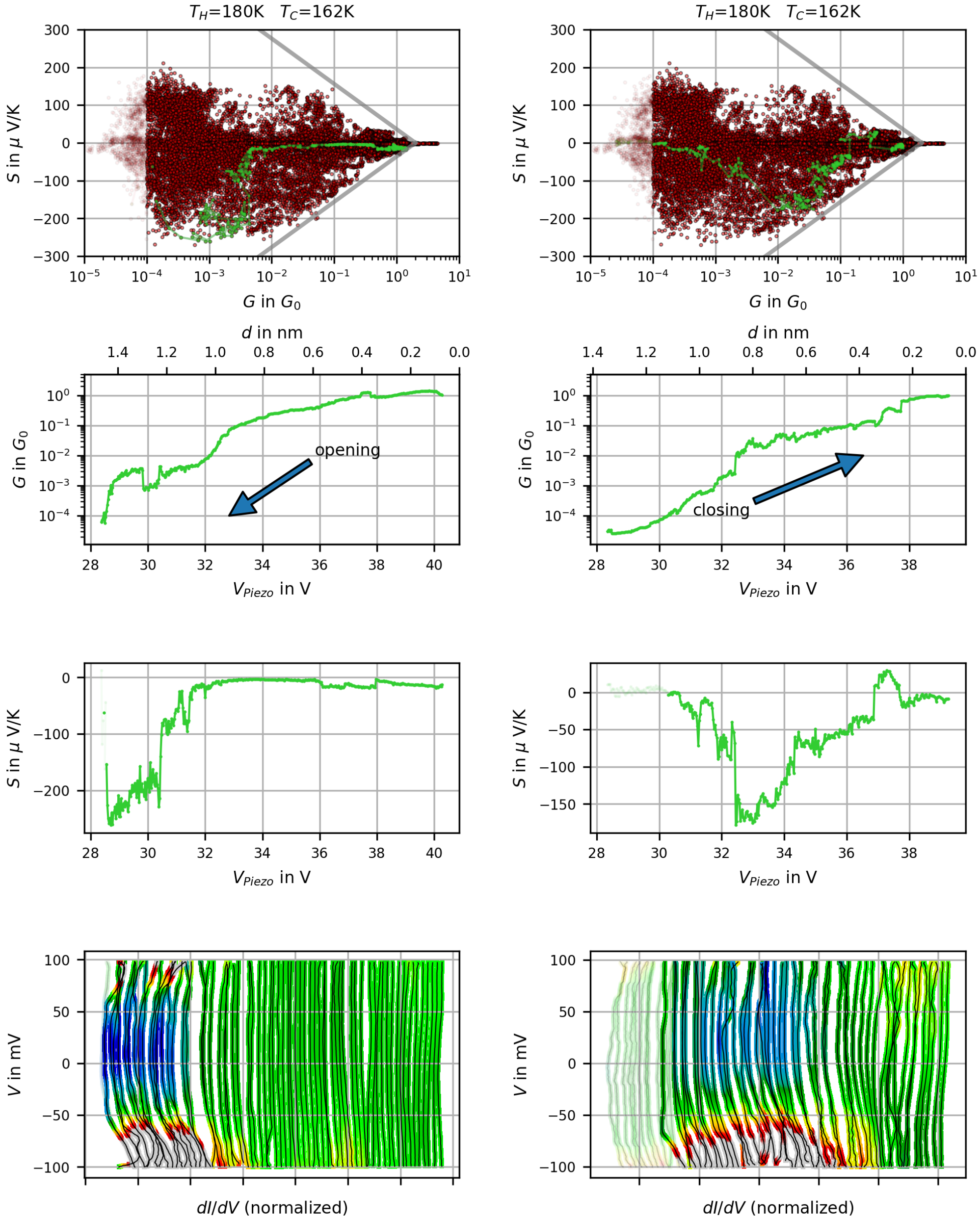

Figure S6: Exemplary opening/closing curves. $n = 5590..6353$

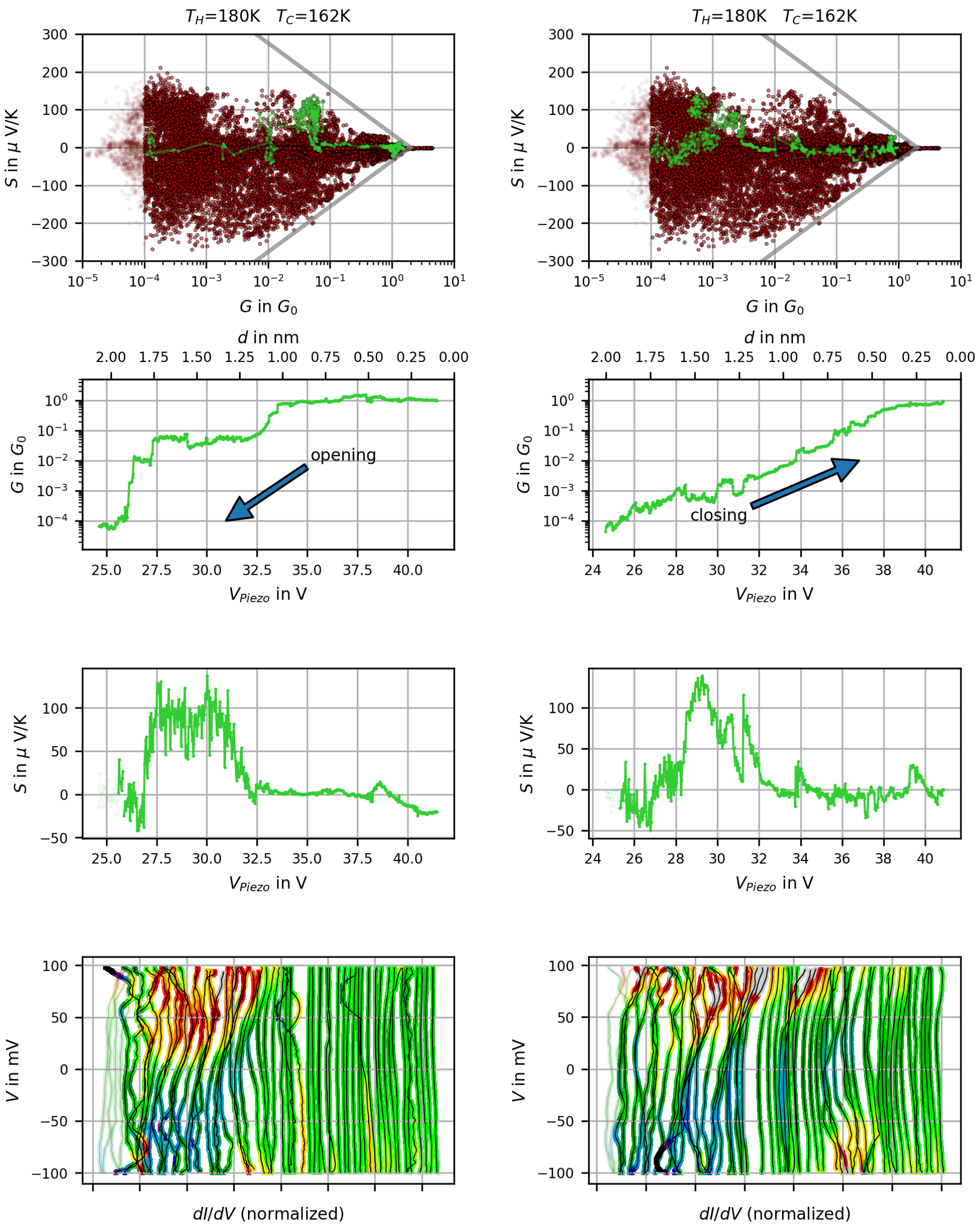

Figure S7: Exemplary opening/closing curves. $n = 10501..11062$

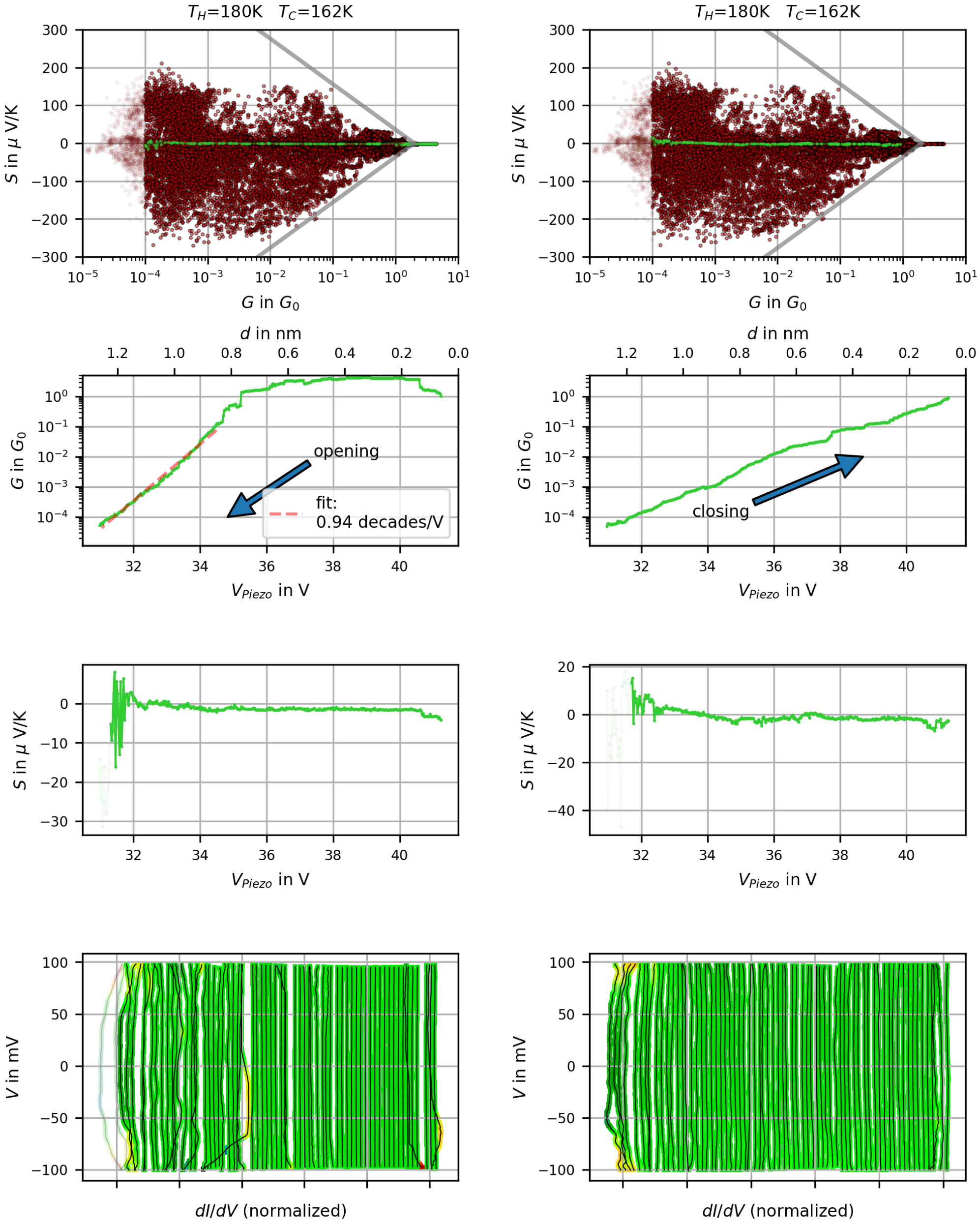

Figure S8: Subset with clean Au-Au contacts used for length calibration. In the opening curve Plateaus at $G > G_0$ are followed by exponential decay in the tunneling regime. $n = 20232..20920$

7 Molecules

Figure S9 shows the molecule which was used. It is a fullerene end-capped molecule that comprises two styrene units that are covalently connected via a [2,2'] paracyclophane moiety in the center 4. The Molecules were drop casted from 10^{-4} molar solution in carbon disulfide solvent. After a droplet of the solution was placed on the chip it was immediately blow dried with nitrogen. The synthesis was performed by Agustin Molina-Ontoria and Nazario Martin, Departamento de Quimica Organica, Facultad de Quimica, Universidad Complutense, E-28040 Madrid, Spain

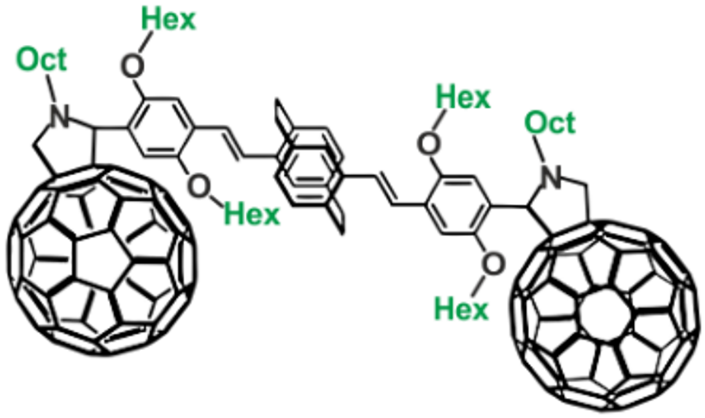

Figure S9: Drawing of the used molecule

8 Electrical measurement circuit

Figure S10 shows a circuit diagram of the electrical measurement setup. In $I(V)$ mode the relay (type: coaxial Rheed) is closed. Voltage is sourced by a the DAQ card and additionally measured by a voltage amplifier. Current is preamplified by an I-V converter and recorded with an analog input channel of the DAQ card.

In thermovoltage mode the relay is opened such that the junction is essentially in an open-contact configuration. Thermovoltage is preamplified by the voltage amplifier and recorded with the DAQ card. The source measure unit (SMU) compensates for input offset currents produced by the voltage amplifier. According to the datasheet of the FEMTO DLPVA-100-F the input bias current is 1 pA with a drift factor of 2.3 / 10°C. It warms up to 40°C in steady state operation. In experiment we find offset currents up to 15 pA. These can be compensated for

with the following routine: (1) The SNJ is completely opened. (2) The relay is closed at 0V output of the DAQ card. (3) The relay is re-opened and a time trace of the open-contact voltage V_{oc} is recorded. This results in a measured voltage increasing linearly with time due to junction and cable capacitance being charged by the offset current. (4) The compensation current sourced by the SMU is adjusted such that the slope of the linear increase is minimized. With this method we can compensate for offset currents with an accuracy on the order of a few pA. At a junction conductance of $10^{-4}G_0$ an offset current of 2 pA would induce an error of $\Delta V_{th} \approx 200\ \mu$V translating into $\Delta S = 10\mu\mathrm{V}/K$ at $\Delta T = 20$ K. Additionally at small conductance the time constant of the V_{oc} measurement increases. Therefore we consider only measurements with $G > 10^{-4}G_0$.

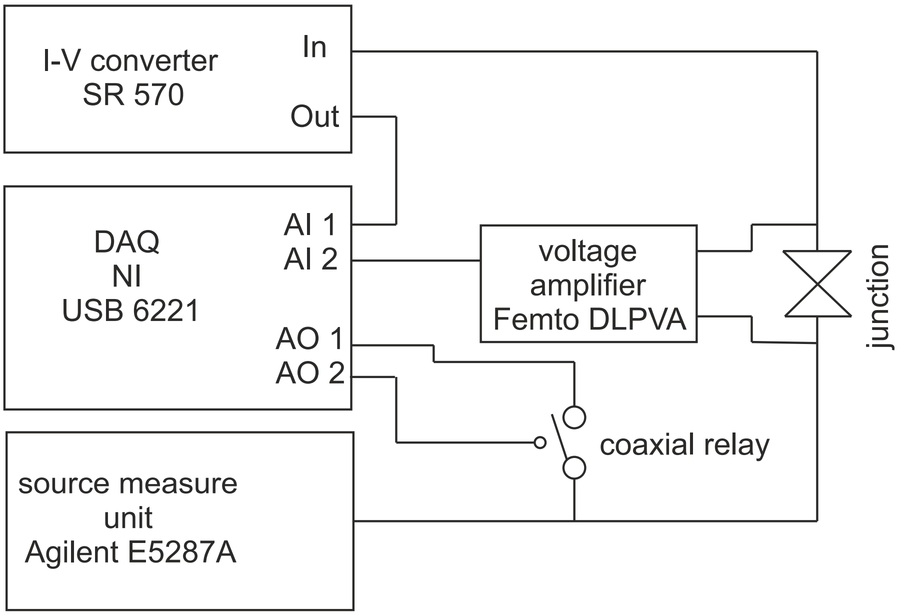

Figure S10: Circuit diagram of electrical measurement

9 In-depth discussion of the anecdotic trajectory in FIG. 1 (b),(d)

With the same method as described in [4] we derived model parameters E_0, Γ, α and G_{off} for each $I(V)$ curve of a subset of a closing trajectory FIG 1 (b). The evolution of parameters as well as modeled $\mathrm{d}I/\mathrm{d}V$ curves are shown in Figure S11. This subset corresponds to the left part of the trajectory in Fig. 1 (d) starting from $(G \approx 10^{-3}\ G_0, S \approx -200\ \mu\text{V/K})$, reaching $(G > 10^{-2}\ G_0, S \approx 0)$ We discuss the parameter evolution during closing of the junction in the following V_{Piezo} intervals:

28.5 V $< V_{Piezo} <$ 29.5 V: Conductance increases an order of magnitude upon closing. At the same time Γ increases by an order of magnitude. Γ values far below $k_BT \approx 15$ meV indicate that the broadening of $\mathrm{d}I/\mathrm{d}V$ is mainly dominated by thermal smearing. The shape of $\mathrm{d}I/\mathrm{d}V$ remains roughly constant in this interval. This then corresponds to $E_0 \approx const.$. G_{off} is vanishingly small. At $V_{Piezo} = 29$ V the $G-S$ pair is close to the excluded area (lower right of FIG. 1 (d)). Negative S correspond to electron-like transport, i.e. E_0 above the Fermi energy.

29.5 V $< V_{Piezo} <$30.8 V: This interval shows the transition from near-resonant transport to resonant transport. The $\mathrm{d}I/\mathrm{d}V$ peak moves towards zero bias voltage. This directly indicates that E_0 moves towards the Fermi energy. Γ remains approximately constant in this interval but still G increases (due to shifting E_0). Slightly below $V_{Piezo} = 30.5$ V the $\mathrm{d}I/\mathrm{d}V$ peak is finally close to 0V coinciding with $E_0 \approx 0$ and S close to zero.

30.8 V $< V_{Piezo} <$31 V: The $\mathrm{d}I/\mathrm{d}V$ peak has moved to positive bias values. Since also the Seebeck coefficient has positive values this is a clear indication that the energy level has now moved below the Fermi energy, also shown by the fit parameters. Note that without having measured the Seebeck coefficient the situation would have been ambiguous, since also α could have changed (stronger coupling to other electrode instead of shifted energy level). Here, also G_{off} has a relevant value. This indicates that other channels contribute to charge transport resulting in small Seebeck coefficients even if the near-resonant $\mathrm{d}I/\mathrm{d}V$ peak would suggest higher absolute values for S.

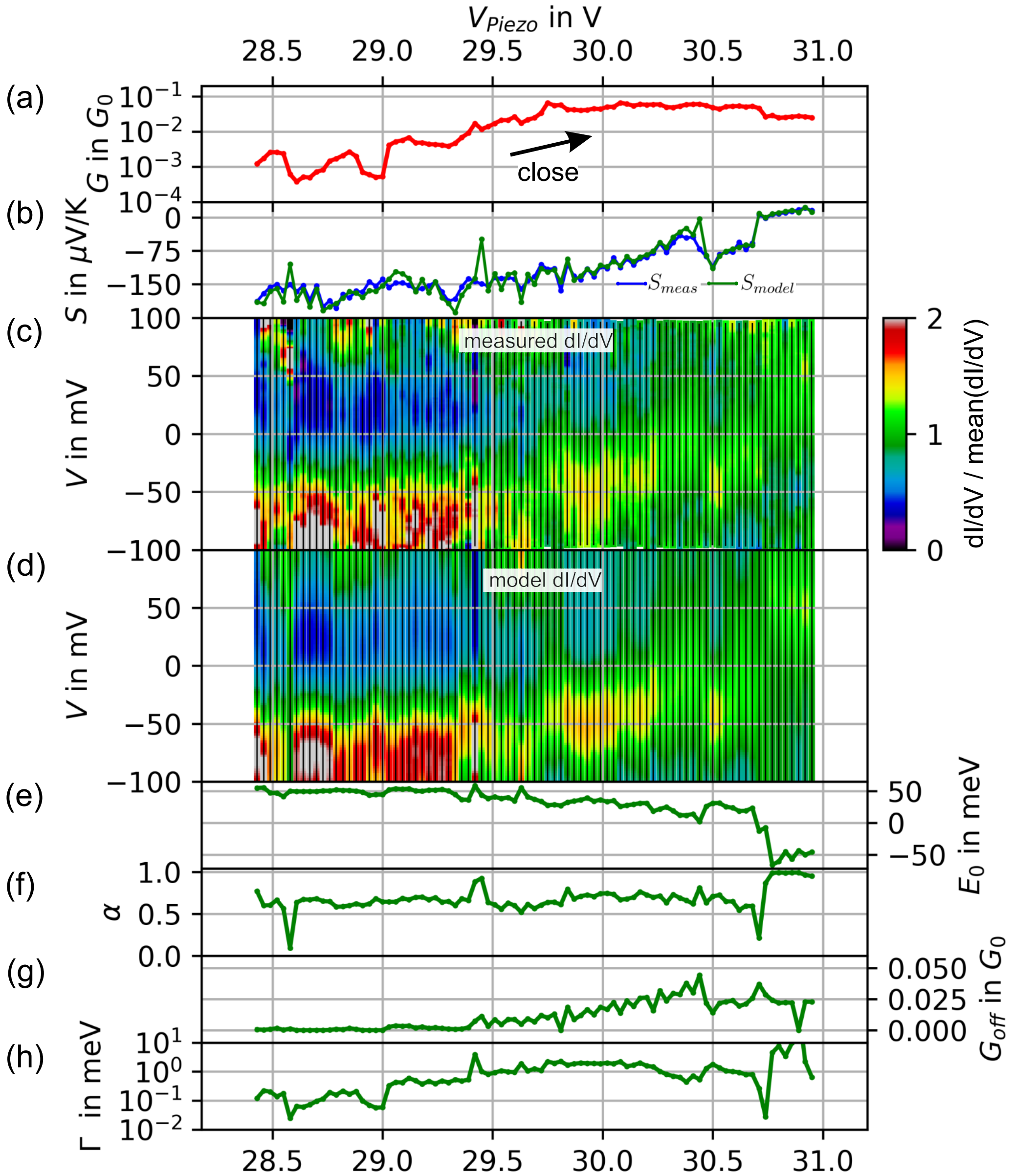

Figure S11: Parametrization of the resonant level model for a subset of FIG. 1 (b) of the main manuscript. Method adopted from [4]. (a): Conductance closing curve, (b): measured Seebeck coefficient S_{meas} and Seebeck coefficient derived from model parameters S_{model}, (c) measured $\mathrm{d}I/\mathrm{d}V$ values. Normalization has been performed as in the main manuscript (values divided by mean value for each curve) for better visibility of curve's shape. (d) $\mathrm{d}I/\mathrm{d}V$ curves calculated with fitted parameters, (e)-(h) fit parameters.

References

[1] Schmutzler, T. *et al.* n-Hexanol Enhances the Cetyltrimethylammonium Bromide Stabilization of Small Gold Nanoparticles and Promotes the Growth of Gold Nanorods. *ACS Applied Nano Materials* **2**, 3206–3219 (2019). URL https://pubs.acs.org/doi/10.1021/acsanm.9b00510.

[2] Cuevas, J. C. & Scheer, E. *Molecular Electronics*, vol. 1 of *World Scientific Series in Nanoscience and Nanotechnology* (WORLD SCIENTIFIC, 2010). URL https://www.worldscientific.com/worldscibooks/10.1142/7434.

[3] Klöckner, J. C., Siebler, R., Cuevas, J. C. & Pauly, F. Thermal conductance and thermoelectric figure of merit of C 60 -based single-molecule junctions: Electrons, phonons, and photons. *Physical Review B* **95**, 245404 (2017). URL http://link.aps.org/doi/10.1103/PhysRevB.95.245404.

[4] Popp, M. A. & Weber, H. B. An ultra-stable setup for measuring electrical and thermoelectrical properties of nanojunctions. *Applied Physics Letters* **115**, 083108 (2019). URL http://aip.scitation.org/doi/10.1063/1.5116673.

[5] Popp, M. A. & Weber, H. B. All data, including raw data is available in an open access repository under DOI 10.22000/321. URL https://doi.org/10.22000/321.

[6] Untiedt, C. *et al.* Calibration of the length of a chain of single gold atoms. *Physical Review B - Condensed Matter and Materials Physics* **66**, 854181–854186 (2002).
